# Correlation between multiple cerebral aneurysms and a rare type of segmental duplication of the middle cerebral artery

**DOI:** 10.1186/s12883-019-1588-8

**Published:** 2020-01-04

**Authors:** Nebojša N. Stojanović, Aleksandar Kostić, Radisav Mitić, Luka Berilažić

**Affiliations:** 1 Clinic for Neurosurgery, Clinical Center, Zorana Đinđića 48, Niš, 18000 Serbia; 2 Faculty of Medicine, University of Niš, Zorana Đinđića 48, Niš, 18000 Serbia

**Keywords:** Duplication of the middle cerebral artery (DMCA), Multiple cerebral aneurysms, Anatomical variations of the middle cerebral artery

## Abstract

**Background:**

Connection between the duplication of the middle cerebral artery (DMCA) and the presence of multiple aneurysms has been described in a small number of cases.

**Case presentation:**

The presence of a rare type of DMCA associated with cerebral aneurysms was diagnosed in 56 year old woman after a rupture of an aneurysm on the dorsal segment of the DMCA. .. The presence of equal diameters of branches of the DMCA and anterior cerebral artery (ACA) could be recorded as trifurcation of the carotid internal artery (ICA). However, due to the anastomosis of the DMCA branches in the area of the M2 segment, the recorded anatomical change represented a segmental duplication of MCA. Three aneurysms that were directly related to the segmental DMCA were diagnosed.

**Conclusions:**

Anatomical variation by type of segmental DMCA is a rare subtype of DMCA. The presence of multiple aneurysms associated with this type of anatomical variation in MCA indicates their high hemodynamic instability.

## Background

**Fig. 1:**
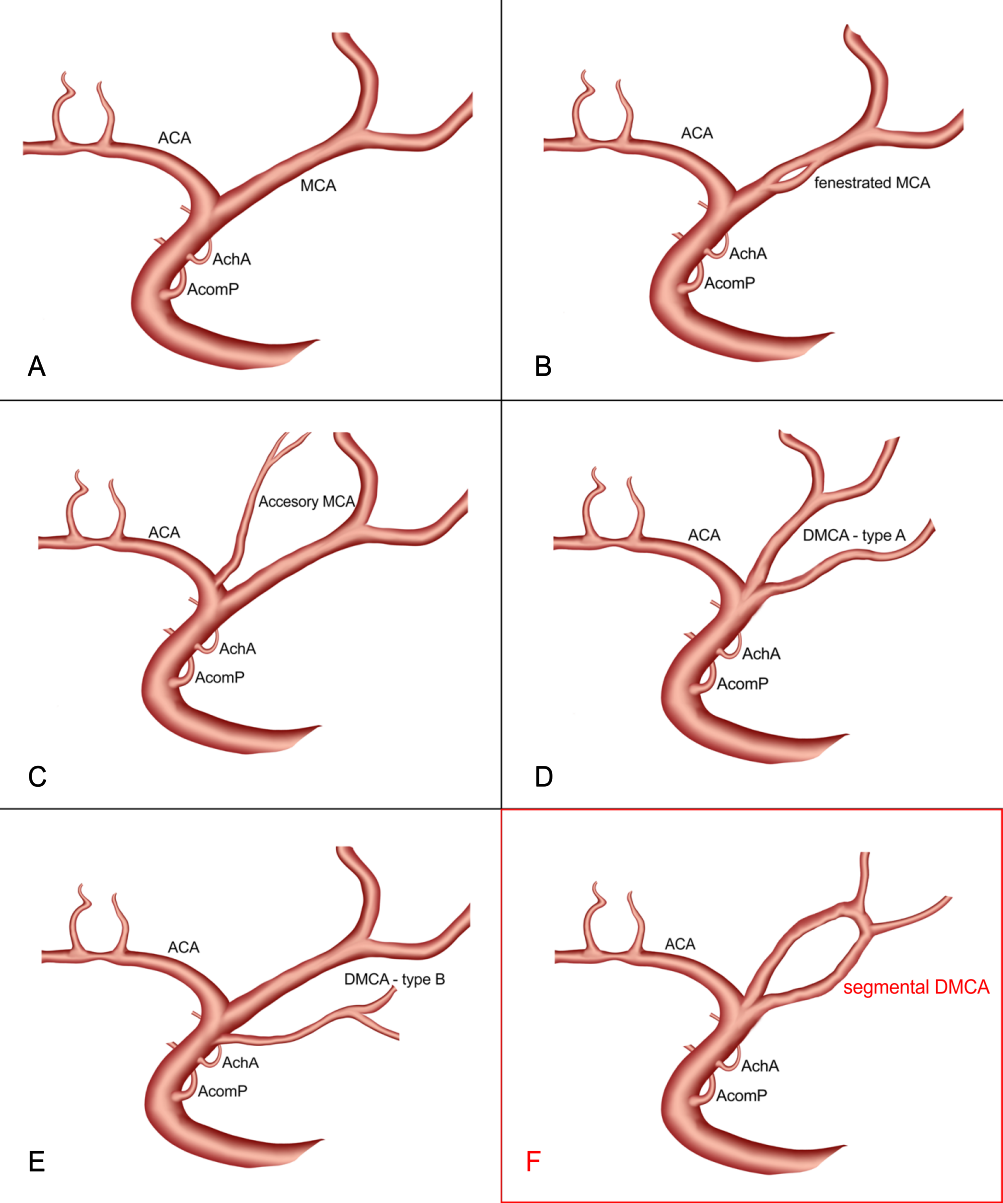
Anatomical variations of the MCA: **a** MCA; **b** fenestrated MCA; **c** accesory MCA; **d** DMCA-type A; **e** DMCA-type A; **f** segmental DMCA

Anatomical variations of the middle cerebral artery (MCA) are significantly less common than those of other intracranial arteries [1, 2]. The most frequent anatomical variations of the MCA are duplication of the middle cerebral artery (DMCA), accessory middle cerebral artery (accessory MCA) and fenestration of the middle cerebral artery (MCA fenestration) (Fig. 1). The incidence of DMCA ranges from 0.7 to 2.9% in autopsy studies [3] and 0.24 to 1.5% in angiographic studies [1, 4]. In 1962, Crompton was among the first to report the existence of MCA anomaly, such as duplication of the MCA, accessory MCA and fenestration of the MCA [5]. In 1973, Teal gave a classification of anomalous changes on the MCA [6]. He defined the term DMCA and the concept of accessory MCA. Under DMCA, he defined two blood vessels originating from the distal end of the internal carotid artery (ICA), and the accessory MCA is defined as an artery arising from the proximal A1 segment of the anterior cerebral artery, just after its origin. The accessory MCA perfuses the territory of the orbito-frontal branch or the perforating arteries (Fig. 1). The incidence of accessory MCA ranges in autopsy and angiographic studies from 0.3 to 4% [2, 5, 6]. The presence of MCA fenestration, i.e., a bridged opening in the blood vessel itself is also very rare and occurs in angiographic studies as 0.17–0.43% [7] and in autopsy studies from 0.02 to 1% [2, 5]. The DMCA was classified into two types, Type A and Type B. Type A refers to the duplication of the middle cerebral artery which separates from the ICA bifurcation, and Type B refers to the blood vessel that arises from the ICA between the anterior choroidal artery (AChA) and bifurcation of the internal carotid artery [1, 4, 5, 8]. Its embryonic origin is still an open question. Yamamoto [8] suggests that the DMCA is a variant of normal MCA bifurcation.

The presence of aneurysms directly related to anatomic variation by type of DMCA is rarely described, and they have an extremely high frequency of rupture [7, 9].

## Case presentation

**Fig. 2:**
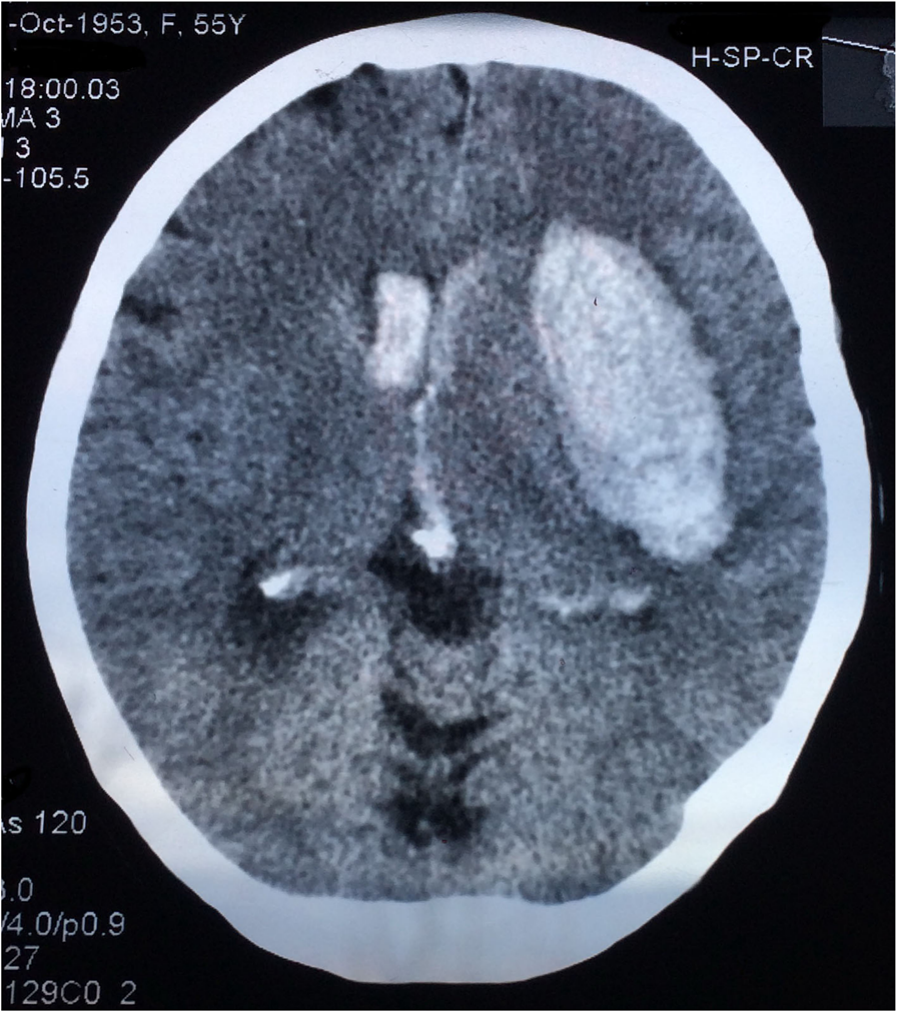
MSCT finding

**Fig. 3:**
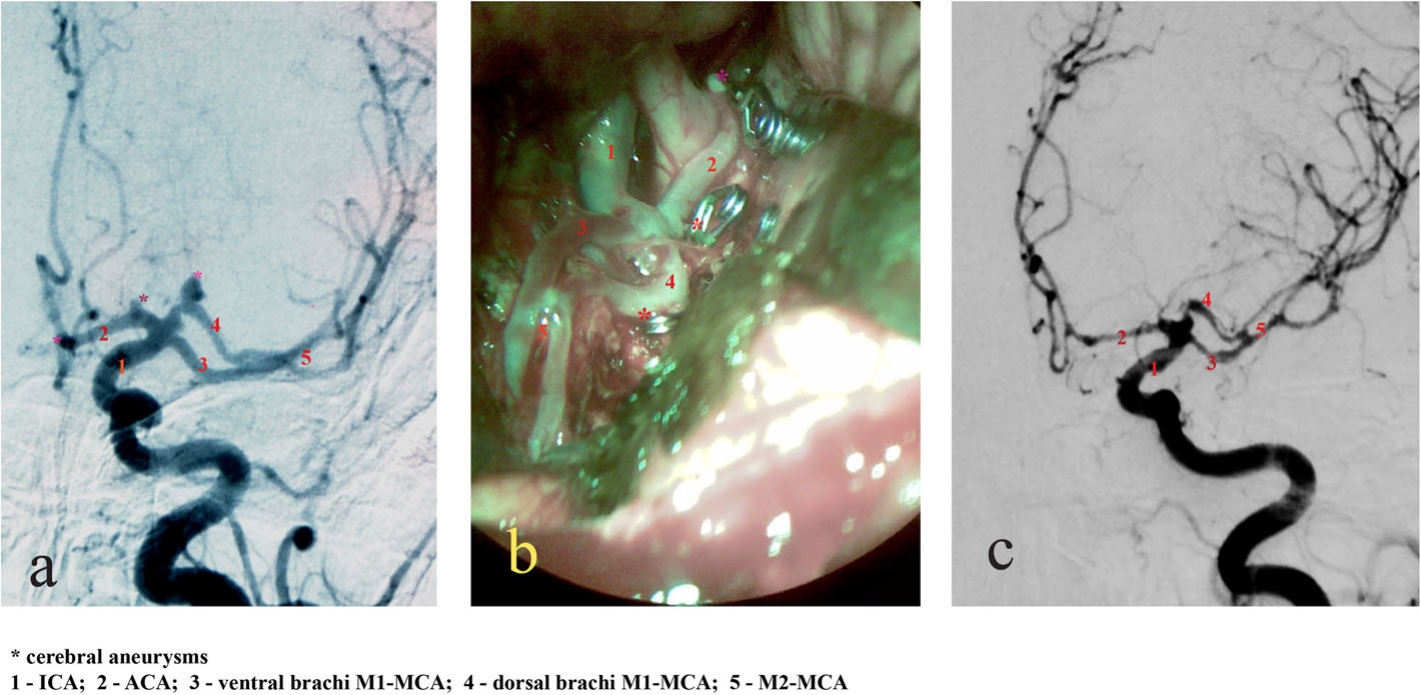
Angiographic and intraoperative finding. **a** Preoperative angiography, **b** intraoperative image after clipping aneurysms, **c** postoperative angiography

A 56-year-old woman was admitted as an emergency, after a sudden loss of consciousness. The multislice CT (MSCT) of the brain performed in a local medical centre showed the presence of intracerebral hematoma 7x4cm in the temporobasal region, with the penetration of blood into the venticular system (Fig. 2). On admission, her Glasgow Coma Scale (GCS) score was 8 with right-side hemiplegia and sensorimotor aphasia was observed. Urgent angiography revealed a duplicated left MCA, which merged in the area of the M2 segment and then immediately divided in the dorsal and ventral branch. In the dorsal branch of the MCA duplication, 6x4mm aneurysm was detected. In addition, in the area of the ICA bifurcation itself, between the dorsal branch of the DMCA and the origin of the left ACA, a 4x3mm aneurysm and another aneurysm on the distal part of the left ACA were detected (Fig. 3a). Emergency surgical intervention was performed by using the pterional approach to the left, with the opening of the Sylvian fissure. The intraoperative finding was in correlation with the angiographic finding. A clear MCA duplication was found, with both branches of the MCA and the initial segment of the ACA being of almost identical diameters (Fig. 3b). The branches of the DMCA merged at the level of the insular segment of the MCA and, immediately thereafter, the normal branching of the MCA in the dorsal-frontal and ventral-temporal branches was found. In the central part of the dorsal branch of the DMCA, the 6x4mm ruptured aneurysm was located. It had a perforating branch arising from the neck. Two smaller aneurysmal changes were observedone in the ICA bifurcation area between the initial segment of the ACA and the dorsal branch of the DMCA, and another one on the distal part of the ACA (Fig. 3b). All three aneurysms were clipped. At the very origin of the ICA bifurcation, in the area between the MCA duplications, a caruncule was observed with the perforating branches arising from it. This lesion was wrapped. The intracerebral hematoma was removed. A second angiogram was performed 7 days after surgery, the aneurysms were occluded but there was evidence of vasospasm (Fig.3c). In the postoperative period, recovery of consciousness occurred with a preoperative neurological deficit in the form of hemiplegia and motor aphasia. After stabilization, the patient was transferred to a rehabilitation center. Examination performed after 6 months showed hemiparesis and aphasia.

## Discussion and conclusions

The DMCA, as we know, is classified into two types. Type A is diagnosed when branches are separated from the very tip of the ICA together with the ACA, and Type B is diagnosed when one branch occurs between the top of the ICA and the onset of AchA [5, 6]. The frequency of type B is twice as common as type A. Since in our case, the DMCA and ACA branches have almost identical diameters, it could be said that this is actually ICA trifurcation [1]. However, one of the peculiarities of our case is the presence of anastomosis between the duplicated MCA branches at the point where the MCA branching would normally occur (Fig.3). This type of anastomosis between duplicated branches of MCA has not been described. Another finding is that the DMCA branches are directly detached from the ICA and both have the length of the entire M1 segment of the MCA. These fused duplicate MCA vassels cannot be categorized as a MCA fenestration. It has been observed that the perforating branches are separated from both parts of the MCA, which supports the notion of real doubling of MCA [2]. Therefore, we have called this type of anatomical variation of MCA - segmental a duplication of M1-MCA (segmental DMCA) (Fig. 1f).

The association between the duplicated MCA and brain aneurysms has been described in 32 cases. Of these, 65% were detected after previous rupture of the brain aneurysm (9). If we know that the presence of cerebral aneurysms in the general population is between 5 and 9% and their rupture occurs in 10 out of 100,000 [10], then the fact that their rupture occurs in 65% of cases of cerebral aneurysms associated with the existence of DMCA indicates a high degree of hemodynamic instability at the point of development of anomalous MCA.

The specificity of our case is that the segmental DMCA is associated with the presence of multiple brain aneurysms that are directly related to the anatomical variation itself. A ruptured aneurysm located in the middle segment of the dorsal branches DMCA indicates an extraordinary haemodynamic load in that area. Providing adequate perfusion in the parts of the brain supplying MCA through two anomalous blood vessels directly leads to increased hemodynamic stress on their walls. In addition, the wall structure of the anomalous DMCA segments will have greater sensitivity to increased perfusion requirements, which in the first part leads to aneurysmal extensions, and later to their rupture.

The association between segmental DMCA with multiple aneurysms and their rupture points to high hemodynamic instability of such anatomical variation.

## Data Availability

All data and material supporting our findings are contained within the manuscript.

## Abbreviations

ACA: Anterior cerebral artery
accessory MCA: Accessory middle cerebral artery
AChA: Anterior choroidal artery
DMCA: Duplication of the middle cerebral artery
ICA: Internal carotid artery
MCA: Fenestration:fenestration of the middle cerebral arter
MCA: Middle cerebral artery
MSCT: Multislice computer tomography
